# The PI3K/Akt/mTOR Pathway: Immuno-Metabolic Orchestration in IR/MASH-Associated Hepatocellular Carcinoma

**DOI:** 10.7150/ijbs.120657

**Published:** 2025-09-27

**Authors:** Jian Zhao, Yuehua Zhang, Zhigong Wei, Kai Li, Lei Sun, Dan Li, Yongsheng Wang

**Affiliations:** 1 Thoracic Oncology Ward, Cancer Center, and State Key Laboratory of Biotherapy, West China Hospital, Sichuan University, Chengdu, Sichuan, 610041, P.R. China.; 2 West China School of Public Health and West China Fourth Hospital, Sichuan University, Chengdu, Sichuan 610041, P.R. China.; 3 Department of Biotherapy, Cancer Center and State Key Laboratory of Biotherapy, West China Hospital, Sichuan University, Chengdu, Sichuan 610041, P.R. China.; 4 Institute of Respiratory Health, Frontiers Science Center for Disease-related Molecular Network, and Precision Medicine Research Center, Precision Medicine Key Laboratory of Sichuan Province, West China Hospital, Sichuan University, Chengdu, Sichuan 610041, P.R. China.

**Keywords:** Hepatocellular Carcinoma, PI3K/Akt/mTOR Signaling Pathway, Insulin Resistance, MASH, Tumor Immune Microenvironment, Metabolic Reprogramming

## Abstract

Insulin resistance (IR) and Metabolic Dysfunction-Associated Steatohepatitis (MASH) are key drivers of hepatocellular carcinoma (HCC), yet the mechanisms underlying their induction of an immunosuppressive tumor microenvironment (TME) require elucidation. This review posits that the PI3K/Akt/mTOR signaling pathway acts as the central integrator of this process, becoming fundamentally rewired—or “imprinted”—by the unique pathological context of IR/MASH-HCC. We highlight how this “imprinted” pathway integrates disparate pathological signals to precisely direct tumor metabolic reprogramming, TME immune landscape remodeling, and the metabolic-dependent regulation of immune cells. We particularly dissect the synergistic amplification of pathway-mediated immune evasion (including PD-L1 upregulation and EMT) by the IR/MASH microenvironment. This integrated framework, which conceptualizes the pathway as the central processing unit of a uniquely aggressive immuno-metabolic phenotype, not only illuminates the unique biology of IR/MASH-HCC but also provides new insights and a theoretical basis for the clinical translation of targeting the PI3K/Akt/mTOR pathway—encompassing novel combination strategies and biomarker development—to foster more effective clinical interventions.

## Introduction

Hepatocellular Carcinoma (HCC), comprising 80-90% of primary liver cancers, is a major cause of cancer-related mortality worldwide, with a particularly poor prognosis in its advanced stages 1. Although viral hepatitis remains the leading global cause, the incidence of HCC associated with metabolic disorders is rising dramatically in industrialized and westernized regions, posing a formidable challenge 2,3. Metabolic dysfunction-associated fatty liver disease (MAFLD), a term that has replaced the former nomenclature of non-alcoholic fatty liver disease (NAFLD), has a global prevalence of approximately 25% and has become the primary cause of chronic liver disease and HCC in many countries 4-6. MAFLD can progress to Metabolic Dysfunction-Associated Steatohepatitis (MASH), a condition accompanied by inflammation and fibrosis that is a high-risk state for cirrhosis and HCC 7. Insulin resistance (IR), a core pathophysiological feature of MAFLD and type 2 diabetes (T2DM), is also a confirmed independent risk factor for HCC 8-10, contributing to a pro-tumorigenic “oncogenic microenvironment” through multiple pathways, including hepatic lipotoxicity, chronic low-grade inflammation, oxidative stress, accelerated fibrosis, and direct stimulation of proliferative pathways 11,12. This confluence of metabolic insults establishes a uniquely pro-tumorigenic landscape, yet a central question remains largely unanswered: How does a systemic metabolic disease like IR translate into a specific, aggressive, and immune-evasive cancer phenotype at the molecular level? This review posits that the answer lies with a single, indispensable protagonist: the Phosphoinositide 3-kinase (PI3K)/Protein Kinase B (Akt)/mammalian Target of Rapamycin (mTOR) signaling pathway, which serves as the critical nexus between systemic metabolic dysfunction and the local tumor immuno-metabolic landscape.

The pathogenesis of HCC is a complex process involving the dysregulation of several key signaling networks, and pathways such as Wnt/β-catenin, MAPK, and Notch are undeniably critical drivers in a broader context 13,14. However, this review asserts that the PI3K/Akt/mTOR pathway occupies a unique and central position specifically within the etiology of MASH-driven HCC. Its centrality is not arbitrary; it is based on a unique convergence of qualifications that no other oncogenic pathway possesses. First, unlike its counterparts, the PI3K/Akt/mTOR cascade is a primary downstream effector of insulin and insulin-like growth factor (IGF) signaling 15, positioning it as a direct molecular “sensor” for the hallmark metabolic disturbances of MASH—namely, IR and the resultant chronic hyperinsulinemia 10. While other pathways contribute to cell proliferation, the PI3K/Akt/mTOR pathway uniquely translates these specific systemic metabolic insults into direct pro-tumorigenic action within the hepatocyte. Second, its downstream effects are profoundly dual-faceted, directly shaping the two core pillars of MASH-HCC progression. On one hand, it orchestrates the profound metabolic reprogramming essential for cancer cell survival, including the enhancement of the Warburg effect and potent stimulation of *de novo* lipogenesis (DNL) 16,17. On the other hand, it is intricately linked to the regulation of the immune landscape, modulating the expression of immune checkpoints like PD-L1 18,19 and actively shaping an immunosuppressive tumor microenvironment through its metabolic outputs 20. Therefore, in the specific subtype of HCC that arises from a backdrop of metabolic disease, the PI3K/Akt/mTOR pathway functions as a central coordinator at the center of a “perfect storm.” It is simultaneously: 1) The Sensor, directly activated by the disease's defining feature; 2) The Engine, driving the metabolic reprogramming required for tumor growth; and 3) The Conductor, orchestrating the immunosuppressive TME through metabolic warfare and checkpoint regulation. It is this unparalleled role as the principal integrator of metabolism and immunity that justifies its selection as the central focus of this review, offering the most relevant lens through which to understand and potentially target MASH-driven HCC.

Despite its importance, few existing reviews have systematically elucidated from an integrated perspective how the PI3K/Akt/mTOR pathway, within the specific pathological context of IR/MASH, acts as a central coordinator to precisely orchestrate the complex “immuno-metabolic-signal” interaction network. Clarifying this unique perspective is crucial for understanding the pathological mechanisms of IR/MASH-related HCC, identifying therapeutic targets, and guiding clinical strategies.

To address this critical gap, this review will comprehensively depict the central coordinating role of this pathway. We will delve into its “molecular imprinting” in the IR/MASH context, its role in driving metabolic reprogramming and shaping the TME, the “metabolic-dependent” regulation of immune cells, key feedback loops, and the synergistic amplification of immune evasion mechanisms. Furthermore, based on these mechanisms, we will analyze the molecular remodeling of the HCC therapeutic response, assess the challenges, and propose novel precision combination therapy strategies. This work therefore aims not only to synthesize existing data but to provide a novel, multidimensional molecular framework for understanding the unique biology of IR/MASH-driven HCC and to offer a theoretical basis for developing more precise and effective therapeutic strategies.

Ultimately, by reframing the PI3K/Akt/mTOR pathway not as a simple linear cascade but as a centrally 'imprinted' immuno-metabolic coordinator, this review provides a unifying framework to reconceptualize the pathogenesis of MASH-driven HCC. It moves beyond a mere catalog of individual molecular events to construct an integrated logic that explains the disease's unique biological aggressiveness and immune resistance. This perspective is therefore not merely academic; it offers a fundamental basis for overcoming current therapeutic challenges and for the rational design of next-generation combination strategies tailored to this growing and distinct patient population.

## The PI3K/Akt/mTOR Pathway: A Double-Edged Sword of Physiological Regulation and Pathological Dysregulation

The PI3K/Akt/mTOR signaling cascade is a highly conserved and vital intracellular network in eukaryotic cells. It integrates extracellular signals (e.g., growth factors like insulin, IGF-1) and intracellular cues (e.g., nutrients, energy status) to precisely regulate a wide range of activities including metabolism, growth, proliferation, survival, migration, and angiogenesis 16,21. Its core components include the lipid kinase PI3K, the serine/threonine protein kinase Akt (also known as PKB), and mTOR, which forms two functionally distinct complexes: mTORC1 and mTORC2 22,23. Physiologically, this pathway is a key mediator of insulin signaling, essential for maintaining glucose homeostasis 9,24. The tumor suppressor Phosphatase and tensin homolog (PTEN) is the most critical negative regulator of the pathway, terminating the signal by dephosphorylating PIP3 25,26. Notably, PTEN's activity is itself precisely regulated by the cellular redox state, with its active site cysteine residue (Cys124) being highly sensitive to inactivation by reactive oxygen species (ROS), a feature of profound importance in pathological states 27.

However, in the pathological environment of IR/MASH, pathway dysregulation presents a central paradox: despite upstream signaling being impeded by inflammatory and lipotoxic insults that impair insulin receptor substrate (IRS) proteins 28-30, downstream branches of the pathway can bypass this blockade to achieve sustained, aberrant activation. This profound and durable rewiring of the pathway's signaling logic, we term 'molecular imprinting'. In this context, molecular imprinting refers to the decoupling of the PI3K/Akt/mTOR pathway from its physiological upstream inputs and negative feedback controls, rendering it constitutively active and hyper-responsive to the specific pathological cues of the MASH microenvironment. A key mechanism of this imprinting is the sustained functional inhibition of PTEN by ROS or lipid peroxidation products 31,32. Concurrently, the pathway's internal negative feedback loops, such as the classic S6K→IRS1 loop, are “hijacked” by pro-inflammatory signals. This decoupling of upstream signal blockade and downstream sustained activation, driven by the failure of negative feedback mechanisms, is the core feature that defines its molecular 'imprinting' 33.

In HCC tumor cells, this pathway's aberrant activation is even more prevalent and complex. The driving factors now include not only the aforementioned environmental factors from MASH but also a host of cell-intrinsic genetic and epigenetic alterations, such as loss-of-function mutations in the *PTEN* gene itself 34, mutations in other pathway components, and the overexpression of upstream receptor tyrosine kinases (RTKs). Furthermore, specific inflammatory-oncogenic circuits reinforce this sustained activation. For instance, in the context of obesity-driven HCC, the pro-inflammatory cytokine IL-6, in concert with androgen receptor signaling, can induce the expression of the cell cycle-related kinase (CCRK), which in turn establishes a positive feedback loop to drive mTORC1 activation 35. These cell-intrinsic changes, superimposed upon the sustained stimuli from the MASH microenvironment (high insulin/IGF, inflammation, oxidative stress), collectively result in a strong and persistent aberrant activation of the PI3K/Akt/mTOR pathway.

Crucially, this 'imprinted' failure of PI3K pathway feedback is not a static feature but the central dynamic engine that propels the malignant progression from MASH to HCC. This progression can be conceptualized as a disastrous cascade initiated by the imprinted pathway: 1. Initiation (Decoupling and Priming): In the MASH stage, sustained PTEN inhibition locks the PI3K/Akt pathway into a state of chronic, low-intensity activation, relentlessly driving pro-tumorigenic shifts 36. 2. Promotion (Survival, Senescence, and Microenvironment Corruption): Sustained Akt activation confers a survival advantage while paradoxically inducing a pro-tumorigenic Senescence-Associated Secretory Phenotype (SASP) that corrupts the local microenvironment 37-39. 3. Progression (Genomic Instability): The combination of continuous proliferative pressure and a ROS-laden microenvironment creates a perfect storm for the accumulation of DNA damage and mutations 40. 4. Transformation (The Final Hit): This process facilitates the acquisition of critical genetic lesions (e.g., in *TP53*, *CTNNB1*) that liberate a hepatocyte clone from all remaining restraints 41. Thus, the 'imprinted' failure of PI3K pathway feedback acts as the master initiator of this disastrous cascade, sequentially navigating a damaged hepatocyte through survival, microenvironment corruption, and genomic instability, ultimately culminating in malignant transformation. At this point, the PI3K pathway, a 'double-edged sword' for maintaining homeostasis, has been fully tipped to its pathological side, becoming a fatal engine of hepatocarcinogenesis. This transformation, however, does not occur in a vacuum; it is forged by the unique pathological pressures of the IR-remodeled liver microenvironment, which lays the foundation for this profound pathway dysregulation.

## IR-Remodeled Liver Microenvironment: Laying the Foundation for Pathway Dysregulation

By triggering a cascade of systemic and local pathophysiological changes, IR profoundly reshapes the liver's tissue architecture, metabolic homeostasis, and immune balance, thereby constructing a unique, highly pro-tumorigenic microenvironment for PI3K/Akt/mTOR pathway dysregulation. Understanding the key components of this microenvironment is crucial for comprehending how the pathway is “imprinted”.

At the metabolic level, a toxic milieu of lipids and their byproducts directly interferes with PI3K/Akt signaling, laying the primary foundation for its 'imprinting'. The core of IR is the reduced responsiveness of target tissues to insulin, leading to an influx of FFAs into the liver and enhanced hepatic DNL 10,42. The key culprits are excessively accumulated lipotoxic molecules, such as saturated fatty acids (SFAs) 43, ceramides 44, and diacylglycerols (DAGs) 15, which disrupt the PI3K/Akt/mTOR pathway by interfering with IRS function or inhibiting Akt phosphorylation.

At the immuno-inflammatory level, the chronic inflammation of MASH provides a continuous barrage of signals that hijack and sustain PI3K/Akt pathway activity. Lipotoxicity, ROS, and gut-derived factors activate innate immune cells like Kupffer cells, which release pro-inflammatory cytokines such as TNF-α and IL-6 31,45. These factors not only amplify IR but also remodel the immune cell landscape towards an immunosuppressive phenotype, characterized by M2-like TAMs, exhausted Teffs, and an increase in Tregs and MDSCs 45-47.

At the oxidative stress level, a state of severe oxidative stress provides the most direct mechanism for 'imprinting' the pathway by functionally inactivating its key negative regulator, PTEN. ROS in MASH are derived from various sources, including mitochondrial dysfunction and NADPH oxidase (NOX) activation 10,36,48. Critical ROS mediators and lipid peroxidation products can directly oxidize or modify PTEN, while chronic oxidative stress can deplete antioxidant systems, ultimately leading to sustained PTEN inactivation 49.

At the matrix remodeling level, the fibrotic landscape provides a source of potent growth factors that offer another layer of sustained, external activation for the PI3K/Akt pathway. Chronic inflammation and hepatocyte injury activate hepatic stellate cells (HSCs), leading to liver fibrosis 50. This altered physical environment exacerbates immunosuppression through growth factors (e.g., TGF-β, HGF) secreted by activated HSCs 51.

In summary, the MASH microenvironment is not a passive backdrop but an active forger. The convergence of lipotoxic interference, inflammatory hijacking, direct PTEN inactivation by ROS, and sustained growth factor signaling collectively and profoundly 'imprint' the PI3K/Akt/mTOR pathway, transforming it from a physiological regulator into a core pathological engine. Once imprinted, this dysregulated pathway becomes a master conductor, orchestrating a profound reprogramming of tumor cell metabolism, as we will discuss next.

## Pathway-Driven Metabolic Reprogramming and Its Immune Shaping of the TME

In the specific pathological context of IR-driven HCC, the PI3K/Akt/mTOR pathway functions as the central driver of a profound metabolic reprogramming. Its impact extends beyond merely satisfying the tumor's own anabolic demands; it actively remodels the entire functional landscape of the TME by orchestrating the release and consumption of key metabolites. This process can be conceptualized as the tumor leveraging metabolic alterations as a weapon to establish an immunosuppressive niche 52. Pathway activation is particularly prominent in the IR environment, driven by complex factors including the common loss of function of the tumor suppressor PTEN, strong stimulation from persistent hyperinsulinemia/IGF signaling, and even direct pathway activation by high-fat diet-induced Akt palmitoylation, providing a direct metabolic link 53.

Firstly, the activated PI3K/Akt/mTOR pathway promotes the Warburg effect. By stabilizing the key transcription factor HIF-1α, possibly in synergy with MYC, it upregulates GLUT1 and key glycolytic enzymes (e.g., HK2, LDHA) 15. This process is greatly amplified in the MASH liver, where pre-existing hyperglycemia provides abundant substrate, while the hypoxic and chronic inflammatory signals within the MASH liver may synergize with pathway activation to further stabilize HIF-1α, pushing glycolysis to its limit and causing a massive accumulation of lactate in the TME 31.

Secondly, the pathway orchestrates a profound shift in anabolic metabolism, with a particular focus on lipids. A primary effect is the potent drive for *de novo* lipogenesis (DNL) and cholesterol synthesis. By phosphorylating and activating the master transcriptional regulator SREBP1, the pathway upregulates key lipogenic genes like *FASN*, *ACC*, and *SCD1* 54. This pathway-driven DNL is superimposed upon a liver already pathologically burdened by steatosis, creating a far more severe lipotoxic environment. Tumor cells actively secrete these newly synthesized lipids, transforming the microenvironment's metabolic landscape. Specifically, this leads to the release of saturated fatty acids (SFAs), such as palmitate, and monounsaturated fatty acids (MUFAs), like oleate 55. These secreted lipids are potent, differential regulators of immune function. The accumulation of SFAs is known to be directly toxic to effector T cells, inducing a form of cell death known as lipoapoptosis 46. In contrast, these lipids can promote the polarization of macrophages towards an immunosuppressive M2 phenotype 56 and provide fuel for the pro-tumorigenic functions of MDSCs and Tregs, which rely on fatty acid oxidation (FAO) 57,58.

Thirdly, the pathway is deeply involved in remodeling amino acid metabolism. It upregulates key amino acid transporters (e.g., SLC7A5/LAT1, SLC1A5/ASCT2) to enhance the uptake of essential amino acids like glutamine, which supports both rapid proliferation and sustained mTORC1 activation 59,60. However, this increased consumption by tumor cells, combined with the inflammatory environment and the enrichment of M2-like TAMs (with high expression of IDO and ARG1), can lead to the severe local depletion of other critical amino acids, such as tryptophan and arginine 61.

**Figure 1:**
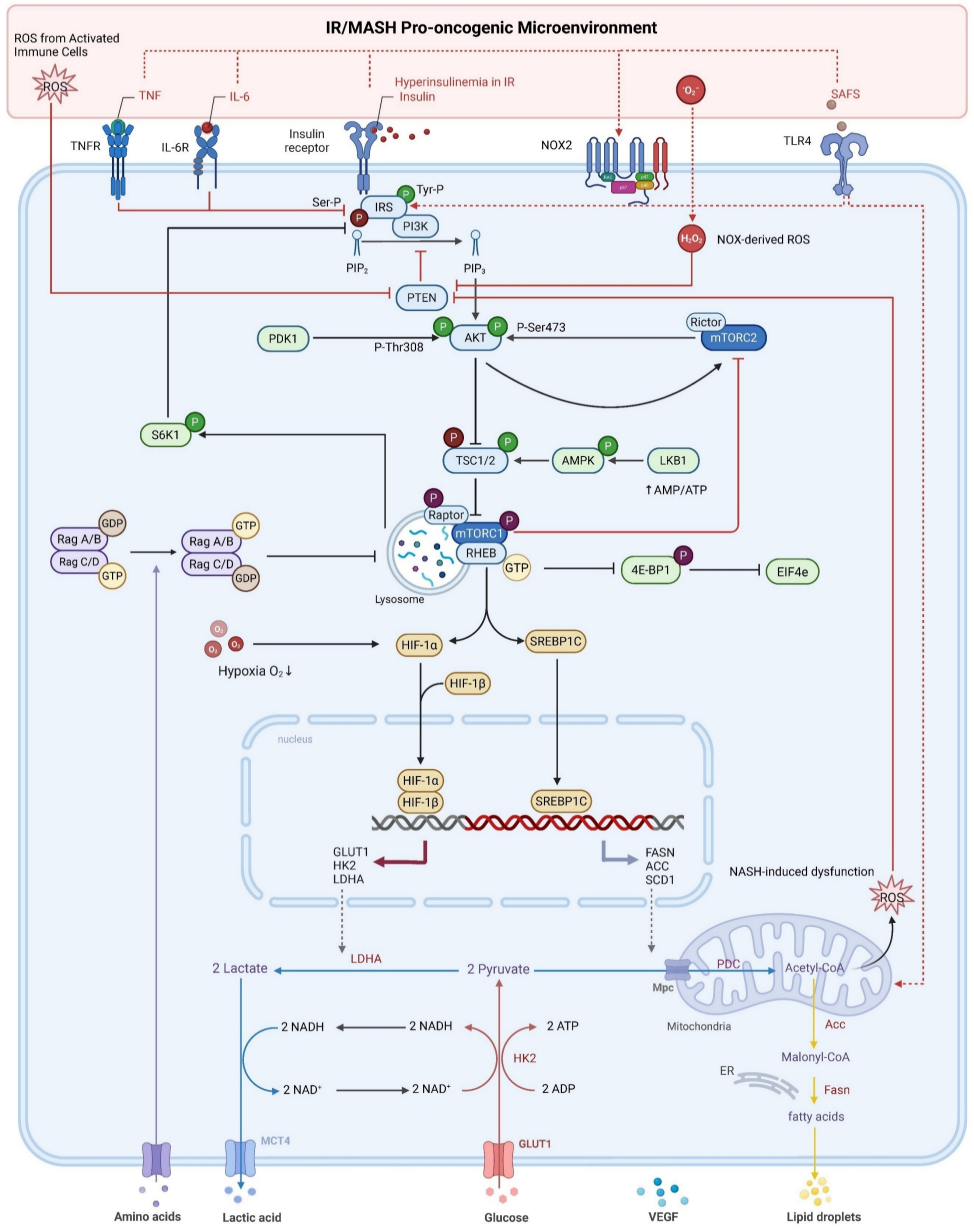
** PI3K/Akt/mTOR pathway dysregulation by the IR/MASH oncogenic microenvironment fuels HCC metabolic reprogramming.** In hepatocellular carcinoma (HCC) cells, the insulin resistance (IR) and **Metabolic Dysfunction-Associated Steatohepatitis (MASH)** microenvironment—characterized by hyperinsulinemia, inflammatory cytokines (TNF-α, IL-6), saturated fatty acids (SFAs), and reactive oxygen species (ROS)—dysregulates PI3K/Akt/mTOR signaling. This occurs via mechanisms including impaired IRS signaling and ROS-mediated PTEN inactivation. These events promote hyperactivation of Akt (by PDK1 and mTORC2) and subsequently mTORC1. Activated mTORC1 drives metabolic reprogramming towards enhanced glycolysis (via HIF-1α) and *de novo* lipogenesis (via SREBP1c), fueling HCC progression and shaping the tumor microenvironment. Phosphorylation is denoted by (P).

These metabolic alterations converge to create a hostile TME through three primary mechanisms. First, resource competition, wherein tumor cells monopolize glucose via the Warburg effect, leading to mTORC1 inhibition and functional exhaustion in effector T cells 46. Second, direct metabolic suppression, where secreted metabolites act as signaling molecules. Lactate, for instance, inhibits T cell function through various means, including cytoplasmic acidification and histone lactylation 62,63. Simultaneously, specific SFAs are directly lipotoxic to these T cells. In stark contrast, these same metabolites—lactate and fatty acids—can fuel the function and polarization of immunosuppressive cells like M2-TAMs and Tregs 47. Third, nutrient deprivation, primarily of key amino acids such as tryptophan and arginine by enzymes like IDO and ARG1, further cripples the anti-tumor T cell response 64 (Figure 1).

Perhaps no single mechanism better exemplifies this coordinated immuno-metabolic crosstalk than the recently identified inflammatory-oncogenic circuit involving the cell cycle-related kinase (CCRK). This circuit acts as a potent upstream activator of mTORC1 signaling in obesity-associated HCC, and is cooperatively induced by obesity-driven pro-inflammatory signals (the IL-6/STAT3 axis) and androgen receptor (AR) signaling—providing a molecular explanation for the male predominance in MASH-HCC. Mechanistically, activated CCRK drives the mTORC1 pathway, which in turn orchestrates a perfect two-pronged assault. On the metabolic front, it amplifies DNL by activating SREBP1. On the immune front, it stimulates the secretion of G-CSF, leading to the robust recruitment of highly immunosuppressive polymorphonuclear myeloid-derived suppressor cells (PMN-MDSCs) into the liver 35. This “inflammatory-CCRK-mTORC1” axis thus represents a powerful molecular bridge that translates the inflammatory and hormonal milieu of MASH directly into coordinated metabolic reprogramming and profound immunosuppression.

## Direct Regulation of Immune Cells by the PI3K/Akt/mTOR Pathway: Metabolic Dependence and Bidirectional Feedback

The influence of the PI3K/Akt/mTOR pathway is not confined to tumor cells; its signaling status within various immune cells in the tumor microenvironment (TME) directly dictates their functional phenotypes, and its activity is itself highly dependent on the local metabolic milieu 60. This “metabolic dependence” of intracellular pathway regulation, coupled with a complex bidirectional feedback network involving other TME components, collectively shapes the immune landscape of IR-driven HCC.

### Metabolic Environment-Shaped Pathway Activity and Functional Choices in Immune Cells

The unique metabolic features of the TME (e.g., low glucose, high lactate, altered lipid profiles) profoundly influence the activity of the PI3K/Akt/mTOR and related pathways (e.g., AMPK) within infiltrating immune cells. For instance, in tumor-associated macrophages (TAMs), lipids enriched in the TME can drive their polarization towards an M2-like, pro-tumorigenic phenotype by specifically activating the internal PI3Kγ signaling pathway 57,65. High lactate may also promote M2 polarization 56. In contrast, the function of effector T cells (Teffs) is highly dependent on mTORC1-driven glycolysis 59,66. The low glucose in the TME directly inhibits mTORC1 activity 46,67, while high lactate can further suppress the PI3K/Akt/mTORC1 pathway 62,68, collectively leading to impaired Teff function and exhaustion 69. This is a key mechanism driving T cell exhaustion, severely limiting their persistence and efficacy (including that of CAR-T cells) in solid tumors 70. In contrast, regulatory T cells (Tregs) exhibit greater metabolic adaptability, such as the ability to utilize lactate 71, allowing them to thrive. This differential response and dependence of various immune cell pathways on the TME's metabolism is a major reason for the formation of the immunosuppressive landscape.

### Feedback Regulation of Tumor Cell PI3K Pathway by TME Cells and Amplification in the IR Context

**Figure 2:**
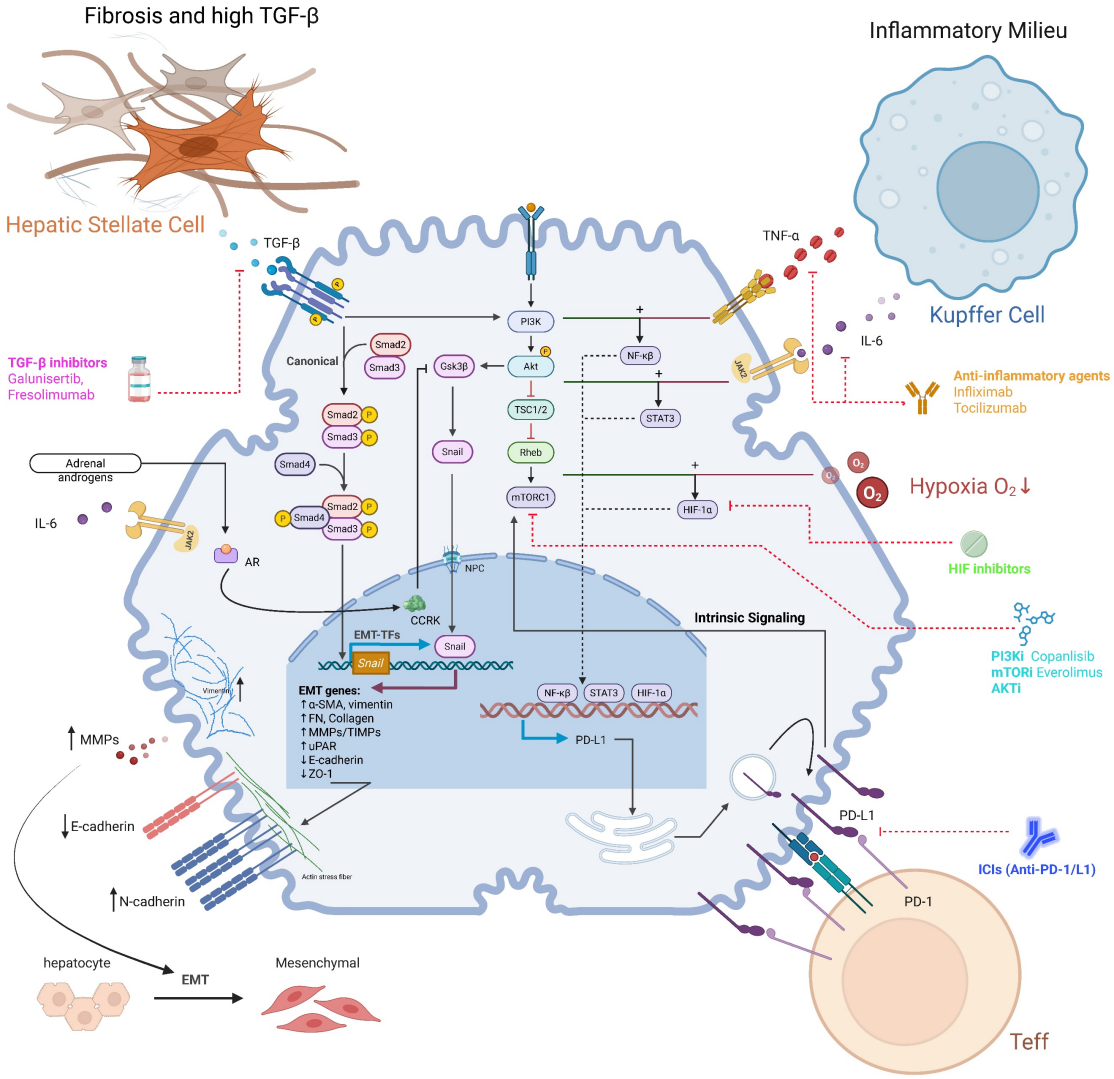
**HCC-shaped metabolic landscape of the TME dictates immune cell fate and function.** HCC cells with activated PI3K/Akt/mTOR signaling actively remodel the tumor microenvironment (TME) by consuming nutrients and releasing metabolites. This creates a metabolically hostile milieu characterized by high lactate, low glucose, lipid accumulation, and amino acid depletion. These distinct metabolic features differentially affect infiltrating immune cells: **Effector T cells (Teffs)** are metabolically crippled by glucose deprivation and lactate/lipid toxicity, leading to exhaustion. **Regulatory T cells (Tregs)** exhibit metabolic flexibility, utilizing lactate and fatty acids to fuel their suppressive functions. **M2-like Tumor-Associated Macrophages (M2-TAMs)** are polarized by lipids and lactate towards a pro-tumorigenic phenotype. **Myeloid-Derived Suppressor Cells (MDSCs)** thrive in the lipid-rich environment, supporting their potent immunosuppressive activities. Collectively, the HCC-orchestrated metabolic reprogramming of the TME creates a deeply immunosuppressive landscape.

Immune and stromal cells in the TME are not passive bystanders; they actively secrete various factors that “feedback” to tumor cells, sustaining the activation of pro-survival/proliferative pathways like PI3K/Akt/mTOR. This feedback is particularly critical in IR/MASH-driven HCC, as the chronic inflammatory and fibrotic environment provides abundant cellular (e.g., activated HSCs, TAMs) and molecular sources for these signals. Key feedback mechanisms include growth factors secreted by activated HSCs and TAMs, such as HGF (activating PI3K/Akt via c-Met) 72 and EGF (activating PI3K/Akt via EGFR and mediating drug resistance) 73. These signals constitute a major external push for maintaining sustained pathway activation in the HSC/TAM-rich IR/MASH environment. Additionally, the inherent chronic inflammation of IR/MASH continuously releases pro-inflammatory cytokines like TNF-α and IL-6 74,75. These cytokines act on tumor cells, activating NF-κB and STAT3 pathways, which can then engage in crosstalk with the PI3K/Akt pathway to synergistically enhance cell survival and proliferation 76,77. (Figure 2). This vicious cycle of metabolic control and pro-tumorigenic feedback creates a pre-conditioned battleground where the tumor's core immune evasion strategies are not just active but are poised for synergistic amplification.

## PI3K/Akt/mTOR Pathway-Mediated Immune Evasion: Synergistic Amplification in the IR/MASH Microenvironment

Aberrant activation of the PI3K/Akt/mTOR pathway within HCC cells orchestrates a complex immune evasion program. In IR/MASH-driven HCC, this evasion appears significantly enhanced, not as a simple consequence of pathway activation, but as a result of a profound “**synergistic amplification**” between pathway-mediated mechanisms and the unique pathological microenvironment (chronic inflammation, hypoxia, fibrosis, etc.) 52,78,79. The IR/MASH microenvironment acts as an active participant, and its unique combination of metabolic and inflammatory pressures interacts with core intracellular signaling pathways to amplify key immune evasion strategies, endowing IR/MASH-HCC with unique “stealth” and “counter-attack” capabilities 80. This section will focus on how two key immune evasion mechanisms—PD-L1 upregulation and epithelial-mesenchymal transition (EMT)—are markedly enhanced by this synergy.

### Synergistic Upregulation of PD-L1: A Convergence of Intrinsic and Extrinsic Signals

The synergistic upregulation of PD-L1 is a prime example of this amplification, driven primarily by the convergence of intrinsic and extrinsic signals. Programmed death-ligand 1 (PD-L1) is a key immune checkpoint that enables immune escape by inhibiting T cell function 81. The PI3K/Akt/mTOR pathway is a core driver of PD-L1 expression in HCC, primarily by activating downstream transcription factors like hypoxia-inducible factor-1α (HIF-1α), nuclear factor-κB (NF-κB), and signal transducer and activator of transcription 3 (STAT3) to promote the transcription of *CD274* (the gene encoding PD-L1) 82. It can also regulate its expression through non-coding RNAs or post-translational modifications.

In IR/MASH-driven HCC, PD-L1 expression is further amplified because the MASH microenvironment's key features—hypoxia and chronic inflammation—are themselves potent external inducers of these same transcription factors. Tissue remodeling and metabolic dysfunction-induced hypoxia directly stabilize and activate HIF-1α 83-85. Simultaneously, chronic inflammation, driven by lipotoxicity, DAMPs, gut-derived LPS, and a plethora of pro-inflammatory cytokines (e.g., TNF-α, IL-6), continuously activates NF-κB and STAT3 pathways in various liver cells 86-88. Consequently, signals from the tumor's intrinsic PI3K/Akt/mTOR pathway converge with signals from the external MASH microenvironment, all activating the same set of critical transcription factors (HIF-1α, NF-κB, STAT3) 89. This multi-source signal convergence, coupled with potential pathway crosstalk (e.g., IL-6/STAT3 and PI3K/Akt interaction) 77 and altered chromatin accessibility 90, leads to a supra-additive amplification of PD-L1 transcription. This synergistically driven high level of PD-L1 dramatically enhances the capacity of IR/MASH-HCC to directly inhibit T cells and evade immune surveillance.

### Synergistic Promotion of EMT: From a Duet to a Self-Reinforcing Loop

The promotion of EMT represents a more complex form of synergy, evolving from a duet of signaling pathways into a self-reinforcing feedback loop. Epithelial-mesenchymal transition (EMT) endows tumor cells with invasive, metastatic, and therapy-resistant properties and is regulated by a network of EMT-inducing transcription factors (EMT-TFs) like Snail, Slug, Twist, and ZEB 91. In HCC, the transforming growth factor-β (TGF-β) pathway and the PI3K/Akt/mTOR pathway are two key upstream drivers of EMT 20,72. The PI3K/Akt pathway promotes EMT by inhibiting GSK3β-mediated degradation of Snail, thereby stabilizing it 92.

In IR/MASH-driven HCC, the EMT process is also significantly enhanced by synergy with the microenvironment, where the key factor is high TGF-β signaling from liver fibrosis. Late-stage MASH is often accompanied by significant fibrosis, where activated hepatic stellate cells (HSCs) are the main source of TGF-β 93. This potent, sustained external TGF-β signal forms a strong synergy with the tumor's intrinsically activated PI3K/Akt pathway, manifested through both crosstalk, as TGF-β can activate PI3K/Akt via its non-SMAD pathways 94, and convergence on the same downstream EMT-TFs 95. This dual-pronged assault results in a more robust and sustained induction of the EMT phenotype.

Furthermore, this synergistic network is amplified by a third crucial element: the intrinsic, pro-tumorigenic signaling of PD-L1 itself. Beyond its well-established role as an immune checkpoint, emerging evidence reveals that tumor cell-intrinsic PD-L1 can function as a signal transducer to directly promote cancer progression 96. In various cancer models, high PD-L1 expression has been shown to directly activate the PI3K/Akt pathway 97. Mechanistically, PD-L1-activated Akt then promotes EMT through the canonical pathway: it phosphorylates and inhibits glycogen synthase kinase 3β (GSK3β), thereby preventing the degradation of the master EMT transcription factor, Snail 98. Crucially, this pathway does not operate in a single direction. A bidirectional regulatory relationship exists where key EMT-TFs, such as ZEB1 and TWIST1, can bind to the PD-L1 promoter region, upregulating its transcription 99,100. This establishes a potent positive feedback loop (“PD-L1 → PI3K/Akt → EMT → PD-L1”), which locks the tumor into a highly aggressive, metastatic, and simultaneously immune-evasive state, further amplifying the pro-invasive signals from both the fibrotic microenvironment and the tumor's intrinsic PI3K/Akt dysregulation.

### Other Synergistic Mechanisms and an Integrated Perspective

Beyond the synergistic enhancement of PD-L1 and EMT, other immune evasion mechanisms mediated by the PI3K/Akt/mTOR pathway may also be amplified in the unique pathological environment of IR/MASH. A compelling example is the direct recruitment of potent immunosuppressive cells. As previously detailed in Section 4, the inflammatory-driven CCRK-mTORC1 axis provides a compelling example of direct immunosuppressive cell recruitment, wherein mTORC1-stimulated G-CSF secretion leads to the robust influx of polymorphonuclear myeloid-derived suppressor cells (PMN-MDSCs) into the liver, actively constructing an immune-evasive niche 35. Other potential synergistic interactions may include pathway-driven VEGF secretion, which, superimposed on the high TGF-β background and hypoxia 101 of the MASH environment, collectively constructs a more complex immunosuppressive cytokine milieu. The chronic metabolic stress of the IR/MASH environment may also select for tumor clones that are more dependent on PI3K/Akt survival signals, reinforcing their resistance to apoptosis 11. As for the net effect of autophagy regulation on immune evasion, it is more complex and requires further investigation.

**Figure 3:**
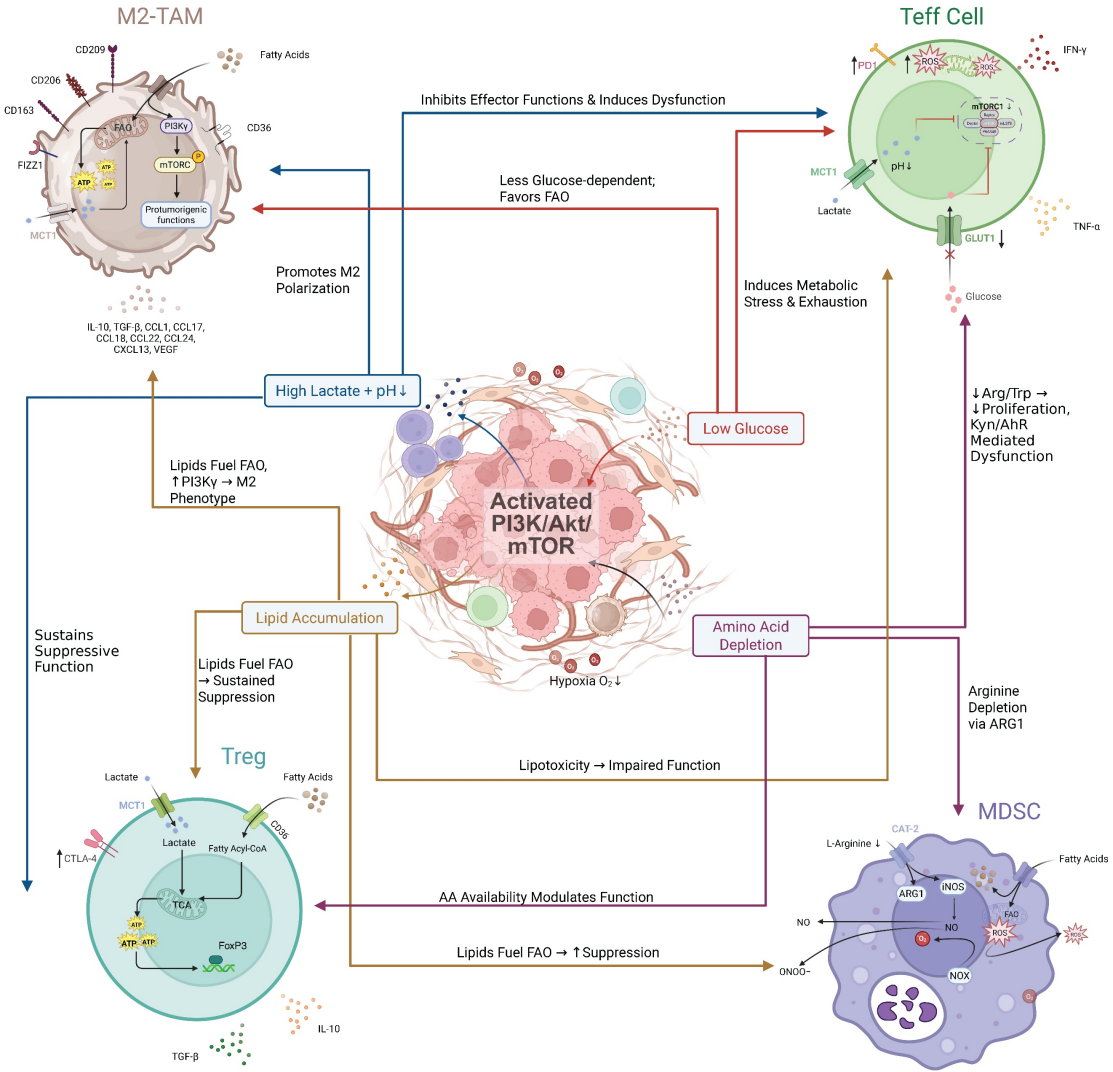
** Synergy between intrinsic PI3K/Akt/mTOR signaling and the IR/MASH microenvironment amplifies immune evasion and reveals therapeutic targets.** In HCC cells, intrinsic PI3K/Akt/mTOR signaling converges with extrinsic cues from the IR/MASH microenvironment to drive robust immune evasion. **(A) Synergy in PD-L1 Upregulation:** The MASH microenvironment, characterized by an **inflammatory milieu** (e.g., TNF-α, IL-6) and **hypoxia**, synergizes with the PI3K/Akt/mTOR pathway to enhance the activity of transcription factors (NF-κB, STAT3, HIF-1α), leading to supra-additive upregulation of PD-L1 and subsequent Teff inhibition. **(B) Synergy in EMT Promotion:** The **fibrotic MASH microenvironment**, rich in TGF-β, activates the canonical SMAD pathway. This synergizes with the PI3K/Akt pathway's stabilization of the EMT-TF Snail, driving a robust EMT program. This network is further amplified by PD-L1 intrinsic signaling and the **inflammatory-oncogenic CCRK axis**, which is co-activated by IL-6 and **androgens via the androgen receptor (AR)**. **(C) Therapeutic Opportunities:** This complex network reveals multiple points for therapeutic intervention, including **PI3K/mTOR inhibitors**, **ICIs**, **anti-inflammatory agents**, **HIF inhibitors**, and **TGF-β inhibitors**, highlighting the potential for mechanism-based combination strategies.

In summary, the unique microenvironment of IR/MASH is not merely a passive background but actively engages in profound synergistic interactions with the aberrantly activated PI3K/Akt/mTOR signaling pathway within tumor cells. This synergy, mainly manifested through signal pathway convergence and crosstalk, directly amplifies the intensity and durability of key immune evasion mechanisms, particularly PD-L1-mediated T cell inhibition and EMT-mediated cell phenotype remodeling. This 'double-hit' on the immune system, driven by the unique synergy within the MASH microenvironment, likely underpins the notoriously aggressive phenotype and therapeutic resistance of this HCC subtype 102, posing significant challenges for current immunotherapies (Figure 3).

## Molecular Remodeling of HCC Therapeutic Response by the IR/MASH Microenvironment and Novel Clinical Translation Strategies

The unique molecular mechanisms detailed previously—the “imprinting” of the PI3K/Akt/mTOR pathway and the “synergistic amplification” of immune evasion—are not just of academic interest; they profoundly impact the response to current HCC therapies and provide a clear rationale for the clinical challenges observed in the MASH-HCC subtype.

The clinical significance of the PI3K/Akt/mTOR pathway in hepatocellular carcinoma (HCC) is underscored by its high frequency of dysregulation and its profound impact on patient prognosis. Aberrant activation of this pathway is one of the most common molecular events in HCC, with upregulation of mTOR signaling reported in approximately 40-50% of cases 54,103. While precise prevalence data for the MASH-HCC subtype is still emerging, the pathway's role is strongly implicated, as the core metabolic drivers of MASH, such as insulin resistance and hyperinsulinemia, are potent upstream activators of PI3K/Akt signaling 15. Furthermore, genomic analyses of MASH-related HCC cohorts have identified somatic mutations in key pathway components, including activating mutations in *PIK3CA* and loss-of-function mutations in the tumor suppressor *PTEN 34*. Clinically, activation of the PI3K/Akt pathway is not a benign event; it serves as a notable risk factor for earlier tumor recurrence and is consistently associated with a more aggressive tumor phenotype and poor patient prognosis 17.

Given its central role, the PI3K/Akt/mTOR pathway has been an intensely investigated therapeutic target for HCC 104. However, the clinical journey of its inhibitors has been challenging. Initial enthusiasm was tempered by the disappointing results of large, randomized phase III trials, most notably the EVOLVE-1 study, where the mTOR inhibitor everolimus failed to improve overall survival in patients with advanced HCC who had progressed on sorafenib 105. In stark contrast, a significant survival benefit has been consistently observed in a specific clinical setting: for HCC patients undergoing liver transplantation, an immunosuppressive regimen based on mTOR inhibitors (sirolimus or everolimus) has been shown to improve both overall and recurrence-free survival 106. The current therapeutic rationale has therefore shifted. The PI3K/Akt/mTOR pathway is now recognized as a critical escape route and a key mechanism of acquired resistance to multi-kinase inhibitors like sorafenib 107. Consequently, the focus of ongoing clinical development has moved towards novel, more specific inhibitors and, crucially, towards combination strategies aiming to overcome these resistance mechanisms 104.

### Challenges to the Efficacy and Safety of PI3K/Akt/mTOR Inhibitors

The clinical application of PI3Ki in HCC is inherently challenging, and these challenges are significantly exacerbated in the IR/MASH context where single-agent efficacy is generally limited and often accompanied by significant toxic side effects 104. The reasons for this failure are a direct and predictable consequence of the “molecular imprinting” of the pathway.

Firstly, metabolic disorders mediate drug resistance. Persistent hyperinsulinemia in the IR state is a potent physiological activator of the PI3K/Akt pathway and can directly antagonize the efficacy of upstream PI3K or Akt inhibitors through an “insulin feedback” effect 108. More critically, the pathway has bypass routes that render it insensitive to standard inhibitors. MASH-associated lipotoxicity, such as the saturated fatty acid palmitate (PA), has been shown to activate Akt through mechanisms independent of the classic PI3K-PIP3 axis (e.g., via ZDHHC17/24-mediated palmitoylation), providing a novel explanation for the poor efficacy of PI3Ki in a lipid-rich MASH microenvironment 109.

Secondly, there is an overlap of metabolic toxicity. Common side effects of PI3Ki (especially PI3Kα and pan-PI3K/mTOR inhibitors) include hyperglycemia and hyperlipidemia 110. For example, hyperglycemia was a common adverse event in the VICTORIA study (NCT02730923) evaluating the mTOR inhibitor vistusertib 111. In IR/MASH-HCC patients with pre-existing severe metabolic disorders, the risk and severity of these side effects are likely increased, complicating clinical management and potentially compromising treatment continuity.

### Challenges to the Efficacy of Immune Checkpoint Inhibitors (ICIs)

Although ICI-based combination therapies have significantly improved the prognosis for some advanced HCC patients 112, a growing body of clinical evidence suggests that the IR/MASH-HCC subtype may have a distinct and often poorer response. Subgroup analyses of multiple clinical trials and real-world studies indicate that non-viral/MASH-HCC patients may have worse overall survival (OS), progression-free survival (PFS), and/or objective response rates (ORR) when treated with ICIs compared to viral HCC patients 113,114. The mechanisms of “synergistically amplified immune evasion” discussed in this review provide a solid molecular basis for understanding this clinical challenge, which manifests on multiple levels:

- **Broad Upregulation of PD-L1 and the “PD-L1 Paradox”:** The MASH microenvironment drives widespread PD-L1 expression on tumor and immune cells through lipotoxicity 79, chronic inflammation 115, and hypoxia 116, creating a formidable immunosuppressive barrier. Even IgA+ cells accumulating in the MASH liver highly express PD-L1 117. However, this high expression does not always translate to a good response, as a “PD-L1 paradox” may exist where ICIs could be ineffective or even harmful in this context 118.
- **EMT and Fibrosis-Mediated Immune Exclusion:** The fibrotic, high-TGF-β MASH microenvironment potently drives EMT in HCC 119. EMT is strongly associated with an “immune-excluded” TME phenotype characterized by reduced CD8+ T cell infiltration 120 and the dense fibrotic matrix also acts as a physical barrier, preventing T cell access to tumor cells 121, a major cause of primary resistance to ICIs.
- **Hostile Metabolic Microenvironment:** The MASH-HCC TME, with its high lactate 122 and abnormal lipid accumulation, directly inhibits effector T cell functions while supporting immunosuppressive Tregs and M2-TAMs 123. This renders T cells ineffective even after the PD-1/PD-L1 brake is released.
- **Deeply Entrenched Immunosuppressive Cell Network:** The MASH TME favors the enrichment of multiple immunosuppressive cell populations (M2-TAMs, Tregs, MDSCs) 3,124,125. The recruitment of these cells is actively driven by tumor-intrinsic signaling; for example, as previously noted, the CCRK-mTORC1 axis induces G-CSF secretion, leading to the accumulation of potent T cell-suppressive PMN-MDSCs 35.

### Precision Combination Therapy Strategies for IR/MASH-HCC

Faced with the complex dysregulation of the PI3K/Akt/mTOR pathway and the unique challenges it poses to existing therapies in IR/MASH-driven HCC, developing mechanism-based precision combination strategies is urgently needed. Table 1 provides a structured overview of these strategies, linking them directly to the core pathological mechanisms discussed in this review.

**Combination with Metabolic Modulators: “Un-imprinting the Pathway”.** A promising direction is to combine PI3Ki with drugs that improve systemic IR and the core pathology of MASH, aiming to “un-imprint” the pathway and restore sensitivity. These include novel antidiabetic agents like GLP-1 Receptor Agonists (GLP-1RAs) and SGLT2 Inhibitors (SGLT2i), which show potential in improving MASLD-related liver outcomes 126-129 and may prevent the insulin feedback effect common in PI3Ki therapy 130,131. Another key agent is Metformin, a classic AMPK activator whose potential to inhibit mTOR signaling suggests its value as a combination partner 132,133. However, its effect when combined with ICIs requires cautious evaluation, as some studies have reported associations with poorer outcomes 134. The critical challenge remains the lack of preclinical studies in well-defined IR/MASH-HCC models to validate these synergistic effects.

**PI3Ki + ICIs: Dismantling Coordinated Immune Evasion.** Positioning PI3Ki as a “sensitizer” or “synergistic partner” for ICIs is a core strategy to overcome the “synergistically amplified” immune evasion in IR/MASH-HCC. The rationale is multidimensional. PI3Ki can broadly remodel the immune microenvironment, particularly by targeting PI3Kδ and PI3Kγ isoforms, which are central to regulating immunosuppressive populations like MDSCs and TAMs 104. Given that the IR/MASH-HCC TME is typically enriched with these cells, selective PI3Ki (especially PI3Kδ/γ inhibitors) hold immense promise for dismantling the immunosuppressive landscape and significantly enhancing ICI efficacy 135-138. Despite promising preclinical data, a stark lack of published data directly evaluating these combinations in relevant IR/MASH-HCC models remains a critical evidence gap 139.

**Triple-Drug Strategies: A Forward-Looking Approach.** Given the complexity of IR/MASH-HCC and the potential for deep-seated resistance to dual therapies 104, exploring “triple therapy” by adding a third agent to the PI3Ki + ICI backbone is a logical future direction. The third agent should be selected to target key drivers of the microenvironment, such as anti-TGF-β drugs to overcome fibrosis or HIF inhibitors to counteract hypoxia 121,140. While theoretically attractive for IR/MASH-HCC, these strategies are currently in the conceptual and very early exploratory stages and require rigorous validation in sophisticated preclinical models and the development of biomarkers to guide individualized combination strategies.

## Summary and Outlook

Despite recent progress in HCC treatment, effectively addressing its molecular heterogeneity, particularly in subtypes associated with metabolic disorders, remains a major hurdle for precision oncology 13. IR and its related conditions, MAFLD/MASH, are key drivers of the growing global HCC burden, urgently requiring a deeper understanding of their unique pathogenic mechanisms. This review has focused on the signaling hub PI3K/Akt/mTOR, systematically proposing an integrated view: in IR-driven HCC, this pathway is not merely an independent signaling module but a central coordinator, “imprinted” by the unique IR/MASH pathological microenvironment. It then precisely orchestrates tumor-intrinsic metabolic reprogramming, immune cell functional remodeling, and complex tumor-microenvironment interactions, ultimately driving tumor progression and immune evasion.

This integrated understanding not only reveals the unique biological complexity of IR-driven HCC but also provides new dimensions for future therapeutic strategies. However, the clinical efficacy of existing PI3Ki in HCC, especially in patients with an IR/MASH background, has often been disappointing 105. This is likely because preclinical research and drug development have failed to fully consider the special role of the pathway as a “coordinator” within the complex, “imprinted” IR/MASH microenvironment. These challenges stem from the pathway's significant resilience and compensatory mechanisms 141, the significant metabolic side effects of its inhibition 142, and the specific interferences presented by the IR/MASH environment itself.

Therefore, future research and therapeutic development must move beyond single-target thinking and adopt a new paradigm. We must recognize that MASH-HCC is not just another etiology of liver cancer; it is a distinct biological entity defined by the central, coordinating role of the “imprinted” PI3K/Akt/mTOR pathway. This paradigm shift requires a fundamental re-evaluation of our entire research and clinical development pipeline:

- **Rebuilding Preclinical Models:** To effectively evaluate new strategies, the research framework itself needs innovation. Standard cell lines or simple xenografts are inadequate. The field urgently needs preclinical models that faithfully recapitulate the “perfect storm” of human IR/MASH-driven HCC: systemic metabolic dysregulation (IR), chronic inflammation, fibrosis, and a functional immune system 118.
- **Defining Composite Biomarkers:** Moving beyond single-gene mutation analysis is critical. A true predictive biomarker for MASH-HCC must be a “composite” panel that captures: 1) host IR/MASH status; 2) tumor PI3K/Akt/mTOR pathway “imprinting” features; and 3) TME “synergistic evasion” features 143.
- **Designing Smarter Clinical Trials:** Future clinical trials must prospectively use IR/MASH etiology as a key stratification factor and incorporate co-primary endpoints that measure not only tumor response but also dynamic changes in the metabolic and immune microenvironment.
- **Developing Rational Combination Therapies:** Based on the pathway's coordinating role and synergy with the microenvironment, exploring rational combinations is crucial, including combinations of PI3Ki with ICIs, metabolic interventions, or other microenvironment modulators.

In conclusion, examining the PI3K/Akt/mTOR pathway within an integrated immuno-metabolic-signal framework for IR/MASH-driven HCC not only deepens our understanding of the pathophysiology of this important HCC subtype but also provides a critical theoretical basis and a fresh perspective for overcoming current therapeutic dilemmas. Although the challenges are immense, a deeper investigation into this complex regulatory network and the development of more intelligent intervention strategies hold the promise of finally translating a deep mechanistic understanding into tangible clinical benefits for this large and growing patient population.

## Figures and Tables

**Table 1:** Mechanism-Based Rationale for Combination Therapies in IR/MASH-HCC.

Therapeutic Strategy	Targeted Pathological Mechanism	Core Mechanistic Rationale	Specific Molecular Targets / Processes	Example Combinations	Potential Challenges
PI3Ki + ICI	Dismantle “Synergistic Amplification” of Immune Evasion	Reprogram the immunosuppressive TME; reduce PD-L1 expression; target inhibitory immune cells.	PI3Kδ/γ in Tregs/TAMs; Tumor PD-L1 (synergistically upregulated); T-cell metabolic fitness.	Anti-PD-(L)1 Ab + PI3Kδ/γ inhibitors	Context-dependent effects on PD-L1; managing overlapping toxicities.
PI3Ki + Metabolic Modulator	Reverse “Molecular Imprinting” of the Pathway	Correct the systemic/local metabolic milieu that sustains pathway activation; prevent insulin feedback resistance.	Systemic IR (HOMA-IR); AMPK activation; mTOR signaling; SGLT2.	PI3Ki + GLP-1RAs, SGLT2i, or Metformin	Lack of preclinical data in relevant models; potential for drug-drug interactions.
ICI + TME Modulator	Overcome the Hostile Tumor Microenvironment	Break down physical and metabolic barriers to T-cell infiltration and function.	TGF-β-driven fibrosis/EMT; Hypoxia-driven lipogenesis; MDSC recruitment.	Anti-PD-(L)1 Ab + Anti-TGF-β Ab, HIF-2α inhibitors, or CCR2/5 inhibitors	Identifying dominant suppressive axis in individual patients.
Triple Therapy (PI3Ki + ICI + TME Modulator)	Mount a Multi-pronged Attack on Deep-seated Resistance	Simultaneously attack the core pillars of MASH-HCC: imprinted signaling, immune checkpoints, and key TME drivers.	PI3K pathway, PD-1/PD-L1 axis, AND a key TME driver (e.g., TGF-β, hypoxia).	PI3Kδ/γ inhibitor + Anti-PD-L1 Ab + Anti-TGF-β Ab	Significant risk of cumulative toxicity; complex biomarker development.

## Abbreviations

ACC: Acetyl-CoA Carboxylase
Akt: Protein Kinase B
AMPK: AMP-activated Protein Kinase
AR: Androgen Receptor
ARG1: Arginase 1
CCRK: Cell Cycle-Related Kinase
DNL: *De Novo* Lipogenesis
DAGs: Diacylglycerols
EGF: Epidermal Growth Factor
EMT: Epithelial-Mesenchymal Transition
EMT-TFs: EMT-inducing Transcription Factors
FAO: Fatty Acid Oxidation
FASN: Fatty Acid Synthase
G-CSF: Granulocyte-Colony Stimulating Factor
GLP-1RAs: GLP-1 Receptor Agonists
GLUT1: Glucose Transporter 1
HCC: Hepatocellular Carcinoma
HGF: Hepatocyte Growth Factor
HIF-1α: Hypoxia-Inducible Factor-1α
HK2: Hexokinase 2
HOMA-IR: Homeostatic Model Assessment for Insulin Resistance
HSCs: Hepatic Stellate Cells
ICIs: Immune Checkpoint Inhibitors
IDO: Indoleamine 2,3-Dioxygenase
IR: Insulin Resistance
IRS: Insulin Receptor Substrate
LDHA: Lactate Dehydrogenase A
MAFLD: Metabolic Dysfunction-Associated Fatty Liver Disease
MASH: Metabolic Dysfunction-Associated Steatohepatitis
MCTs: Monocarboxylate Transporters
MDSCs: Myeloid-Derived Suppressor Cells
mTOR: mammalian Target of Rapamycin
mTORC1: mTOR Complex 1
mTORC2: mTOR Complex 2
NF-κB: Nuclear Factor-κB
ORR: Objective Response Rate
OS: Overall Survival
PD-L1: Programmed Death-Ligand 1
PFS: Progression-Free Survival
PI3K: Phosphoinositide 3-kinase
PI3Ki: PI3K/Akt/mTOR inhibitors
PMN-MDSCs: Polymorphonuclear Myeloid-Derived Suppressor Cells
PPARs: Peroxisome Proliferator-Activated Receptors
PTEN: Phosphatase and Tensin Homolog
ROS: Reactive Oxygen Species
RTKs: Receptor Tyrosine Kinases
SASP: Senescence-Associated Secretory Phenotype
SGLT2i: Sodium-Glucose Cotransporter 2 Inhibitors
STAT3: Signal Transducer and Activator of Transcription 3
TAMs: Tumor-Associated Macrophages
Teff: Effector T cells
TGF-β: Transforming Growth Factor-β
TME: Tumor Microenvironment
Tregs: Regulatory T cells
VEGF: Vascular Endothelial Growth Factor
